# Mucosal Fibrosis on Biopsy Predicting Intestinal Perforation in Adult-Onset IgA Vasculitis: A Case Report

**DOI:** 10.7759/cureus.93252

**Published:** 2025-09-26

**Authors:** Shoichi Yoshinaga, Ryota Sakai, Akiko Shibata, Takahiko Kurasawa, Koichi Amano

**Affiliations:** 1 Rheumatology and Clinical Immunology, Saitama Medical Center, Saitama Medical University, Kawagoe, JPN

**Keywords:** biopsy, fibrosis, iga vasculitis, intestinal perforation, pathology

## Abstract

We report a case of adult-onset IgA vasculitis that required surgery due to the perforation of the jejunum despite treatment with high-dose glucocorticoids and resolution of the inflammatory response. A mucosal biopsy before treatment revealed only localized fibrosis, while surgical pathology demonstrated ulceration and perforation with extensive submucosal fibrosis at the same site. These findings suggest that mucosal fibrotic changes observed on biopsy may indicate a risk of perforation and support the early use of cyclophosphamide or other agents for refractory disease.

## Introduction

Immunoglobulin A vasculitis (IgAV), formerly known as Henoch-Schönlein purpura, is characterized by IgA1-dominant immune complex deposition in small blood vessels throughout the body. It frequently involves the joints, skin, kidneys, and digestive tract, leading to severe complications such as progressive glomerulonephritis, gastrointestinal (GI) perforation, and intestinal obstruction. IgAV is less common in adults, with an annual incidence of 0.8-5.1 per 100,000 individuals [1], and its clinical features differ from those in children [2]. Two main differences are identified between adults and children with IgAV; first, bowel perforations or intussusceptions are rare in adults, and second, they are at higher risk of developing significant renal impairment, including end-stage kidney disease [3-4]. However, GI involvement frequency, such as abdominal pain and intestinal bleeding, in adults was relatively high (37-53%) in previous studies [2,5]. The duodenum and small intestine are the most frequently affected organs in IgAV with GI involvement, and abdominal pain is typically intermittent and spasmodic in nature [5]. The classic histological findings of IgAV are leukocytoclastic vasculitis (LCV) and IgA deposition in vessel walls on immunofluorescence staining, which is rarely observed on GI biopsy [6]. Biopsy findings include neutrophilic infiltration within the small bowel and colon, with the duodenum being the most commonly affected [6]. However, no reports have focused on fibrosis in small GI biopsies of IgAV patients, and its clinical significance remains unclear. In this study, we report a case of IgAV that required surgery due to perforation of the jejunum despite treatment with high-dose glucocorticoids and resolution of the inflammatory response. The biopsy of the jejunum revealed a small cluster of fibrosis in a small part of the intestine; however, postoperative pathology revealed extensive submucosal fibrosis. This case suggested that fibrotic lesions in small intestinal biopsies may predict intestinal perforation and indicate the need for treatment with cyclophosphamide or other agents.

## Case presentation

A 33-year-old man presented with abdominal pain and a lower limb rash one month prior to admission. Subsequently, he developed watery diarrhea and melena. Initial laboratory tests at an outside hospital revealed leukocytosis, markedly elevated C-reactive protein (CRP), and computed tomography (CT) evidence of small bowel wall thickening. Despite empirical antibiotic and anti-diarrheal treatments, his symptoms progressed, prompting referral to our institution.

**Figure 1:**
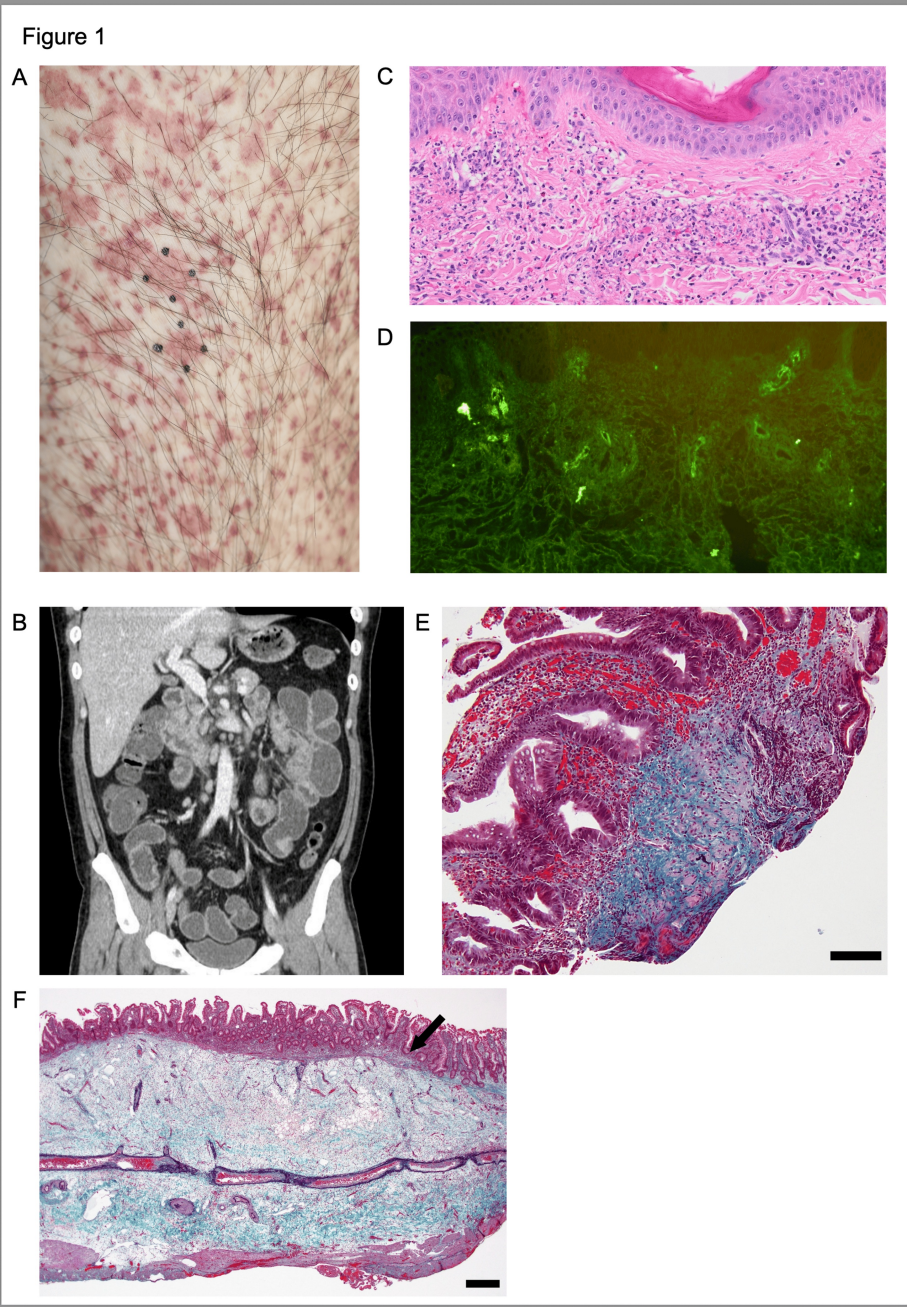
Clinical, radiological, and pathological findings (A) Palpable purpura on the right lower extremity; (B) Contrast-enhanced abdominal computed tomography (Siemens SOMATOM X.ceed 128-slice CT scanner) showing segmental small bowel wall thickening with mucosal hyperenhancement; (C) Histopathological examination of biopsy showing leukocytoclastic vasculitis on Hematoxylin and Eosin (H&E) staining (×200 magnification); (D) Deposition of IgA in the dermal vessels by immunofluorescence (×200); (E) Endoscopic biopsy with Masson's trichrome (MTC) staining reveals mucosal intrinsic layer fibrosis (scale bar:100μm); (F) surgical specimen showing transmural fibrosis extending through the submucosa (black arrow indicates disruption of the muscularis mucosae; scale bar: 1 mm; MTC staining).

**Figure 2:**
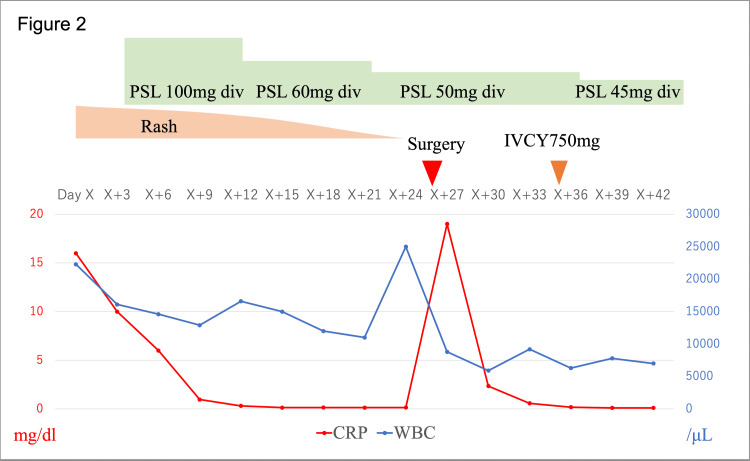
Clinical course of the patient The right vertical axis represents white blood cell count, the left vertical axis represents C-reactive protein levels (mg/dl), and the horizontal axis represents hospital day (days since admission). Treatment timeline, including drug administration (type, dose), and surgical intervention (timing), is noted above the graph. WBC - white blood cell; CRP - C-reactive protein; PSL - prednisolone

On admission, vital signs showed low-grade fever (37.6 °C) and tachycardia (135 bpm). Physical examination demonstrated class I obesity (body mass index 31.4 kg/m^2^; 172 cm, 93 kg), palpable purpura on the trunk and extremities (Figure 1A), and abdominal tenderness without peritoneal signs. Laboratory findings included leukocytosis with neutrophilia, thrombocytosis, hypoalbuminemia, abnormal liver function tests, markedly elevated inflammatory markers (Table 1), immunoglobulin abnormalities (low IgG/IgM and normal IgA), and mild nephropathy (hematuria and proteinuria). All autoantibody panels and blood cultures were negative. Abdominal CT with contrast demonstrated segmental small bowel wall thickening with preserved enhancement (Figure 1B). His past medical history was significant only for gastroesophageal reflux disease, with no relevant family history. Diagnostic workup included skin biopsy, which confirmed LCV with vascular IgA deposition (Figure 1C and Figure 1D), and endoscopy that revealed diffuse erosion, marked neutrophilic infiltration, and focal mucosal fibrosis at the jejunum (Figure 1E). Malignant tumors, infectious diseases, and other vasculitides were ruled out as differential diagnoses. The patient was diagnosed with IgAV, and treatment with intravenous prednisolone (100 mg/day, 0.93 mg/kg/day) and a proton pump inhibitor was initiated on hospital day five, which led to symptom improvement and CRP normalization within five days. However, on hospital day 24, sudden-onset abdominal pain accompanied by a sharp increase in white blood cells and CRP prompted emergency surgery, which confirmed intestinal perforation of the jejunum. Histological examination revealed full-thickness ulceration and perforation with extensive fibrosis and collagen hyperplasia in the subserosal layer at the perforation site. Fibrinoid necrosis and LCV were also observed. The small intestinal mucosa demonstrated extensive fibrotic lesions and disruption of the mucosal muscle layer (Figure 1F). Postoperatively, the patient received intravenous cyclophosphamide pulse therapy (750 mg, biweekly 6 times) with gradual prednisolone taper and was discharged on day 42 (Figure 2).

**Table 1:** Laboratory data on admission WBC - white blood cell; RBC - red blood cell; PLT - platelet; TP - total protein; AST - aspartate aminotransferase; ALT - alanine aminotransferase; ALP - alkaline phosphatase; γGTP - gamma-glutamyl transferase; CK - creatine kinase; LDH - lactate dehydrogenase; BUN - blood urea nitrogen; eGFR - estimated glomerular filtration rate; CRP - C-reactive protein; ESR - erythrocyte sedimentation rate; ASO - anti-streptolysin o; ASK - anti-streptokinase; HbA1c - hemoglobin A1c; TIBC - total iron binding capacity; UIBC - unsaturated iron binding capacity; APTT - activated partial thromboplastin time; PT% - prothrombin time %; PT-INR - prothrombin time international normalized ratio; KL-6 - Krebs von den Lungen-6; C3 - complement component 3; C4 - complement component 4; PR3-ANCA - proteinase 3–anti-neutrophil cytoplasmic antibody; MPO-ANCA - myeloperoxidase–anti-neutrophil cytoplasmic antibody; anti-GBM - anti-glomerular basement membrane antibody; IgG - immunoglobulin g; IgA - immunoglobulin a; IgM - immunoglobulin m; IgE - immunoglobulin e; T-SPOT - t-spot tuberculosis specific IFNγ; TP antibody - treponema pallidum antibody; HBs - hepatitis b surface; HBc - hepatitis b core; HCV - hepatitis c virus; Urine NAG - urinary n-acetyl-β-d-glucosaminidase; Urine β2-MG - urinary β2-microglobulin

Test Item	Value	Lower limit value	Upper limit value	Unit
WBC	22,300	3300	8600	/μl
Neutrophils	85	42.4	75.0	%
Lymphocytes	10.6	18.2	47.7	%
Monocytes	3.7	3.3	9.0	%
Eosinophils	0.4	0.4	8.6	%
Basophils	0.3	0.2	1.4	%
RBC	3.53	4.35	5.55	million/μl
Hemoglobin	9.9	13.7	16.8	g/dl
Hematocrit	30.8	40.7	50.1	%
PLT	546	158	348	1000/μl
Total Protein	5.9	6.6	8.1	g/dl
Albumin	2.1	4.1	5.1	g/dl
AST	42	13	30	IU/l
ALT	53	10	42	IU/l
ALP	258	38	113	IU/l
γGTP	22	13	64	IU/l
Total bilirubin	0.7	0.4	1.5	mg/dl
CK	58	59	248	IU/l
LDH	212	124	222	IU/l
BUN	14	8	20	mg/dl
Creatinine	0.79	0.65	1.07	mg/dl
eGFR	92.0	60	-	ml/min
Sodium	131	138	145	meq/l
Chloride	91	101	108	meq/l
Potassium	4.9	3.6	4.8	meq/l
CRP	16.78	0.00	0.14	mg/dl
ESR	53	2.00	10	mm
ASO	89	0	239	IU/ml
ASK	640	0	1280	x
Glucose	102	73	109	mg/dl
HbA1c	5.9	4.9	6.0	%
Ferritin	376	31	325	ng/ml
Fe	11	2.7	4.6	μg/dl
TIBC	174	40	188	μg/dl
UIBC	163	255	434	μg/dl
APTT	27.7	24.1	31.7	sec
PT%	99.8	74.4	120.0	%
PT-INR	0.98	-	-	
D-dimer	29.3	0	0.99	ng/dl
Rheumatoid factor	4	0	15	IU/ml
Anti-Nuclear antibody	<40	0	39	titer
KL-6	99.4	105.3	401.2	U/ml
C3	166	73	138	mg/dl
C4	47	11	31	mg/dl
PR3-ANCA	1.0	0	3.4	U/ml
MPO-ANCA	1.0	0	3.4	U/ml
Anti-GBM	1.0	0	3	U/ml
IgG	570	861	1747	mg/dl
IgA	322	93	393	mg/dl
IgM	17	33	183	mg/dl
IgE	282	0	173	IU/ml
T-SPOT	negative			
TP antibody	negative			
β-D glucan	negative			
Procalcitonin	negative			
HBs antigen	negative			
HBs antibody	negative			
HBc antibody	negative			
HCV antibody	negative			
Blood culture 2 set	negative			
Urine protein (dipstick)	+1			
Urine occult blood (dipstick)	±			
Urine RBCs	1–4			/HPF
Urine WBCs	10–19			/HPF
Urine protein (quantitative)	65	0	-	mg/dl
Urine creatinine (quantitative)	284	0	-	mg/dl
Urine NAG	39.7	0	5	IU/L
Urine β2-MG	1628	0	150	μg/L

## Discussion

To our knowledge, this case represents the first report to suggest that fibrotic findings on biopsy may serve as risk factors for perforation in patients with IgAV. The presence of intestinal fibrosis on biopsy implies more extensive submucosal fibrosis and potentially reflects longer disease duration, both of which may predispose to perforation. The etiology of IgAV involves a complex interplay among genetic predisposition, particularly in human leukocyte antigen (HLA) class II regions, and environmental triggers, such as infections, that stimulate the autoimmune system [7]. While the predilection for GI lesions in IgAV remains incompletely understood, several genetic factors have been implicated. Polymorphisms in the intercellular adhesion molecule-1 (ICAM-1) gene are linked to a decreased risk of severe GI complications [8]. The frequent GI involvement in IgAV may be explained by Peyer's patches serving as the primary induction sites for intestinal IgA responses, contributing to the higher prevalence of GI lesions in IgAV compared with other vasculitides [5].

Submucosal fibrosis represents a pathological hallmark of Crohn's disease [9]. Chronic inflammation replaces collagen fibers and adipose tissue in the normal submucosal tissue with fibrous tissue, resulting in submucosal shrinkage. In stenotic lesions, fibrosis of the submucosa is prominent, accompanied by submucosal hyperplasia; additionally, it may become thickened enough to obscure the submucosal layer [10]. Current fibrosis scoring systems in inflammatory bowel disease incorporate both imaging data and biomarkers [11]. For Crohn's disease, histopathological scoring of fibrosis has been evaluated by comparing radiographic images and resected specimens [12-13]. Previously published histological scoring indicated that this was a severe or high-grade fibrotic lesion [12-13].

Although pediatric IgAV typically follows a benign course, adult-onset IgAV can lead to severe clinical manifestations and poorer outcomes [14]. GI involvement occurs at similar rates in pediatric and adult cases, but adults are at higher risk of life-threatening complications such as GI perforation [2]. Management of GI involvement in IgAV remains controversial, though steroids and immunosuppressive agents are considered beneficial in severe cases [5]. While mortality in adults with IgAV is not typically linked to GI complications, immunosuppressive agents such as cyclophosphamide may be necessary for patients requiring emergency surgery [5]. In this case, early high-dose steroid therapy initially improved symptoms; however, the subsequent development of GI perforation was unpredictable and necessitated emergency surgery. Notably, the biopsy findings suggested increased perforation risk. Histopathological analysis showed extensive fibrosis, LCV, and vascular changes in the perforated small intestine, consistent with chronic vasculitis and prolonged tissue repair. Similar histological findings occurred in inflammatory bowel diseases, such as ulcerative colitis and Crohn's disease, where the degree of mucosal fibrosis correlates with disease severity [15]. The widespread fibrosis observed in this case suggests a strong inflammatory response and prolonged tissue repair, which may explain the unpredictable perforation. As previously reported [5], a sudden rise in CRP level or new-onset abdominal pain during treatment should prompt suspicion of perforation and warrant immediate imaging studies.

The condition of the patient stabilized after cyclophosphamide pulse therapy, but whether the perforation was prevented by administering cyclophosphamide alone remains unclear. No other treatments without glucocorticoids have been established for IgAV with intestinal lesions. However, cyclophosphamide [16], rituximab [17], and intravenous immunoglobulin [18] have been used in the literature. In addition, intestinal perforation after glucocorticoid therapy has been recognized not only in vasculitides but also in patients with diverticula of the colon according to the literature [19-20]. We cannot rule out that high-dose glucocorticoid administration inhibited tissue repair via the immune response in the intestine, potentially inducing perforation. The presence of fibrosis on biopsy indicates refractory disease and is a sign to reconsider the treatment strategy.

## Conclusions

This case demonstrates adult-onset IgAV complicated by GI perforation, accompanied by massive fibrotic changes despite anti-inflammatory treatment. Mucosal layer fibrosis in the biopsy tissue was the sole predictor of perforation. While the direct relationship between intestinal mucosal fibrosis and perforation remains unclear, the presence of significant inflammatory changes due to vasculitis and subsequent fibrotic alterations, particularly at the perforation site, suggests a potential risk factor for GI perforation. If fibrotic changes are detected on biopsy, glucocorticoids and cyclophosphamide or other agents should be initiated. Further research is needed to elucidate the pathogenesis of GI complications associated with IgAV and its optimal management.
